# Evaluating the pediatric neurology training curriculum in SCFHS-accredited programs: a multi-institutional survey study

**DOI:** 10.1186/s12909-026-09926-w

**Published:** 2026-07-17

**Authors:** Ahmed K. Bamaga, Hebah F. Qashqari, Anas S. Alyazidi, Mohammed M.S. Jan, Raidah S. Albaradie, Khalid J. Hundallah, Ali Al-Otaibi, Ahmed R. Al Rumayyan, Fahad A. Bashiri

**Affiliations:** 1 Department of Pediatrics, Faculty of Medicine, King Abdulaziz University, Jeddah, Saudi Arabia; 2 Department of Pediatrics, King Faisal Specialist Hospital & Research Centre, Jeddah, Saudi Arabia; 3 Department of Paediatric Neurology, The Royal London Hospital, NHS Barts Health, London, UK; 4 Neuroscience Center, King Fahad Specialist Hospital, Dammam, Saudi Arabia; 5 Pediatric Neurology Division, Department of Pediatrics, Prince Sultan Military Medical City, Riyadh, Saudi Arabia; 6 Pediatric Neurology Department, National Neuroscience Institute, King Fahad Medical City, Riyadh, Saudi Arabia; 7 College of Medicine, King Saud Bin Abdulaziz University for Health Sciences, Riyadh, Saudi Arabia; 8 King Abdullah International Medical Research Center, Ministry of National Guard, Riyadh, Saudi Arabia; 9 Division of Pediatric Neurology, Department of Pediatrics, King Abdullah Specialist Children’s Hospital, National Guard Health Affairs, Riyadh, Saudi Arabia; 10 Pediatric Neurology Division, Department of Pediatrics, College of Medicine, King Saud University Medical City, King Saud University, Riyadh, Saudi Arabia

**Keywords:** Pediatric neurology, Graduate medical education, Residency training, Curriculum evaluation, Medical education assessment, Program directors

## Abstract

**Background:**

Pediatric neurology training must evolve to meet increasing clinical complexity and rapid advances in neuroscience while maintaining high standards of family-centered care. In Saudi Arabia, structured residency programs have expanded under national accreditation frameworks; however, systematic evaluations of curriculum adequacy from the faculty perspective remain limited. This study aimed to comprehensively evaluate the pediatric neurology training curriculum within the Saudi Commission for Health Specialties (SCFHS)-accredited training system (including programs in Saudi Arabia and the United Arab Emirates [UAE]) from the faculty perspective, identifying strengths, gaps, and differences between program directors (PDs) and non-director faculty.

**Methods:**

We conducted a multi-institutional cross-sectional study between September and December 2025 targeting pediatric neurology faculty, including PDs, deputy directors, core faculty, and clinical staff involved in resident education. A validated 47-item questionnaire assessed satisfaction across six core curriculum components (educational resources, graduate autonomy and safety, clinical knowledge, clinical–academic balance, research opportunities, and professionalism), identified priority training gaps, and collected qualitative recommendations. Quantitative data were analyzed using descriptive statistics, non-parametric comparisons, correlation analysis, and exploratory multivariable linear regression. Open-ended responses underwent inductive thematic analysis.

**Results:**

Forty-three consultants participated, including nine PDs. Overall satisfaction was moderate, with the highest ratings for resident professionalism (mean 3.8/5) and the lowest for clinical–academic balance (2.8/5) and research opportunities (2.9/5). Genetics and hereditary diseases (74%), neuroimmunology (70%), and neurodevelopmental disorders (63%) were the most frequently cited domains requiring greater emphasis. PDs reported significantly lower overall satisfaction than non-directors (β = −0.37, *p* = 0.02), particularly regarding academic time and research infrastructure. Satisfaction with educational resources strongly correlated with perceived research opportunities (ρ = 0.58, *p* < 0.001). Qualitative themes highlighted excessive service demands, insufficient protected academic time, limited research mentorship, and the need for structured training in rapidly evolving subspecialties.

**Conclusion:**

Pediatric neurology training in the region demonstrates strong clinical and professional foundations but is constrained by systemic challenges in academic balance, research support, and subspecialty preparedness. These perception-based findings suggest that PDs’ heightened concerns may inform future curriculum development. Potential areas for consideration include protected academic time, enhanced research infrastructure, and nationally coordinated curricula in high-priority domains. However, these are exploratory implications requiring further evaluation before implementation.

**Supplementary Information:**

The online version contains supplementary material available at https://doi.org/10.1186/s12909-026-09926-w.

## Background

Pediatric neurology occupies a pivotal role in child health, encompassing a broad spectrum of conditions that collectively represent a leading cause of childhood morbidity, mortality, and long-term disability worldwide, with conditions ranging from epilepsy and neurogenetic syndromes to neurodevelopmental disorders and acquired brain injuries [1]. The complexity of this field necessitates a highly skilled workforce trained not only in sophisticated diagnostics and therapeutics but also in compassionate, family-centered care [2].

Globally, the structure and content of pediatric neurology residency training have undergone substantial transformation. Historically, apprenticeship models and training have progressively formalized into competency-based frameworks, such as the Accreditation Council for Graduate Medical Education (ACGME) milestones in the United States (US) and the CanMEDS physician competency framework adopted in Canada and other regions [3, 4]. This shift emphasizes the balanced development of medical expert, communicator, collaborator, scholar, and professional roles. Concurrently, the rapid pace of discovery in areas such as neurogenetics, neuroimmunology, and neuromodulation demands that training curricula be dynamic and forward-looking [5]. Over the last two decades, competency-based medical education (CBME) has increasingly integrated Entrustable Professional Activities (EPAs) as a practical framework for operationalizing competencies into observable, assessable units of professional work. EPAs provide a link between abstract competency domains and real-world clinical responsibility, allowing multi-level entrustment decisions that reflect readiness for independent practice. Despite the global shift toward EPA-informed curricula, their structured integration within pediatric neurology training curricula in the region remains insufficiently described [6].

In Saudi Arabia, the field of pediatric neurology has experienced parallel growth, shaped by national healthcare transformation under Vision 2030 and the oversight of the Saudi Commission for Health Specialties (SCFHS) since the program’s establishment in 2010 [7]. The SCFHS has established structured residency programs designed to cultivate clinical excellence, research capability, and leadership [8]. According to internal data by the Saudi Pediatric Neurology Society (SPNS), currently, an estimated 150 physicians graduate annually from pediatric neurology residency programs across the Kingdom. The total consultant workforce in pediatric neurology has grown to approximately 112 specialists as of 2024. However, their geographic distribution reveals significant regional disparities where Riyadh hosts 50 consultants, the Western Province 24, the Eastern Province 20, the Southern Province 10, Qassim 6, and the Northern Province only 2. These disparities pose challenges for equitable access to specialized care and for distributed training models that ensure all residents receive adequate exposure to diverse clinical cases and expert mentorship. While this represents significant progress, the unique demographic, cultural, and healthcare system context of the region necessitates a tailored educational approach. Challenges such as geographic distribution of specialists, integration of rapidly advancing subspecialties, and balancing service loads with academic development are particularly salient in the regional training landscape [9].

Despite these advancements, a significant gap persists. There is a paucity of published data systematically evaluating the perceived strengths and weaknesses of the pediatric neurology training curriculum from the perspective of those who deliver it, the faculty and program directors (PDs). While studies from North America and Europe have surveyed training perceptions, their findings may not be fully generalizable to our context due to differences in healthcare infrastructure, resource availability, and disease epidemiology [10]. Multi-institutional studies within the SCFHS-accredited systems have largely focused on workforce numbers or individual program descriptions rather than on a comprehensive, multi-institutional assessment of curriculum content, educational methods, and perceived gaps [11].

Understanding faculty perceptions is crucial, as they directly inform curriculum delivery, resident assessment, and program improvement. PDs, bearing ultimate responsibility for training outcomes, offer a uniquely valuable perspective on systemic strengths and constraints [12]. A rigorous evaluation of the current curriculum is a prerequisite for evidence-based reform, ensuring that the next generation of pediatric neurologists in SCFHS-accredited programs are equipped with the competencies required to meet national health needs and excel in an increasingly complex global medical landscape.

Therefore, this study aims to conduct a multi-institutional evaluation of the pediatric neurology training curriculum in SCFHS-accredited systems from the faculty perspective. Specifically, it seeks to: (1) assess satisfaction with core curriculum components, including educational resources, clinical-academic balance, and research opportunities; (2) identify clinical domains and professional skills perceived as requiring greater emphasis; and (3) explore differences in perceptions between PDs and non-PD faculty members. By elucidating these insights, this research intends to provide a foundational evidence base to guide targeted curriculum development, optimize resource allocation, and ultimately enhance the quality of pediatric neurology training in the region.

## Materials and methods

### Study design and setting

Following the Strengthening the Reporting of Observational Studies in Epidemiology (STROBE), we performed a multi-institutional, cross-sectional convergent parallel mixed‑methods survey. Quantitative (Likert‑scale items) and qualitative (open‑ended free‑text prompts) data were collected simultaneously, analyzed separately (descriptive statistics, regression, and thematic analysis), and then merged during interpretation. The study targeted all SCFHS‑accredited pediatric neurology residency programs. These included programs within Saudi Arabia as well as a program in the United Arab Emirates (UAE) that hold SCFHS accreditation, thereby functioning as part of the unified national training system. Invitations were distributed to all 12 SCFHS-accredited pediatric neurology PDs identified through official SCFHS records and professional networks. Participating institutions included academic medical centers, government hospitals, and specialized tertiary referral centers.

For non-PD faculty, we employed a purposive sampling strategy to ensure representation from all major training institutions within the SCFHS-accredited system. Potential participants were identified through formal records. Invitations were sent to approximately 120–150 pediatric neurologists; a precise denominator could not be determined due to the purposive method, which prioritized institutional representation over a known sampling frame.

A total of 43 physicians completed the survey. No formal sample size calculation was performed before the study, as a census approach was used for PDs, and the study was exploratory in nature (post-hoc power analysis is reported in the Statistical Analysis section). Among the 12 accredited PD positions, one program was still undergoing full accreditation, and one PD was on leave during the study period. Of the 10 eligible and available PDs, 9 responded, yielding a 90% participation rate among accessible PDs and representing 75% of all accredited PD positions. Additionally, 34 non-PD faculty members participated. A formal response rate for this group could not be calculated due to the purposive sampling approach, which prioritized institutional representation over a known sampling frame.

### Participant recruitment and eligibility

The target population consisted of faculty physicians actively engaged in pediatric neurology resident education and supervision. Eligible participants included current PDs, former PDs, deputy PDs, core faculty members, and clinical supervisors. We implemented a purposive sampling strategy for non-PD faculty to ensure representation from all major training institutions across the SCFHS-accredited system. For PDs, a census approach was used, inviting all 12 accredited PDs through SCFHS records. For non-PDs, we systematically identified potential participants through SCFHS records and the Saudi Pediatric Neurology Society directory, which includes formal WhatsApp groups and program leadership nominations. Recruitment was conducted via official channels, society mailing lists, and direct email correspondence. To optimize participation, we sent two follow-up reminder emails at two-week intervals following the initial invitation.

Thus, PDs were sampled by census, while non-PD faculty were sampled purposively to achieve broad institutional representation. Therefore, no overall response rate is reported.

### Survey instrument development and validation

The survey questionnaire was developed through a multi-stage, iterative process to ensure validity and relevance. An initial draft was informed by a comprehensive review of existing literature on competency-based medical education and pediatric neurology training curricula. This draft underwent rigorous review by an expert panel of five specialists in pediatric neurology and medical education, including three current PDs, to assess content validity, clarity, and comprehensiveness. Subsequently, the revised instrument was pilot-tested with a convenience sample of four pediatric neurologists not involved in the expert panel to evaluate item comprehension, estimate completion time, and identify any technical issues. The final 47-item survey was organized into four sections. Section A captured demographic and professional background information (11 items). Section B evaluated perceptions of the current training curriculum using 5-point Likert scales (1 = Very Dissatisfied/Very Inadequate to 5 = Very Satisfied/Very Adequate) and multiple-choice questions (22 items). Section C focused on identifying specific curriculum gaps and training priorities through ranking exercises and open-ended prompts (8 items). Section D solicited open-ended recommendations for curriculum improvement (6 items). The full survey instrument is provided as Supplementary File 1.

### Data collection and management

Data were collected anonymously using a secure, web-based survey platform (Google Forms^®^). The survey link was distributed alongside a detailed cover letter explaining the study’s aims, its voluntary nature, and guarantees of confidentiality. No personally identifiable information was collected at any point. Upon closure of the data collection window, responses were downloaded into a structured Microsoft Excel spreadsheet (Microsoft Corp.) for initial cleaning, coding, and organization. Qualitative data from open-ended questions were transcribed verbatim for subsequent analysis.

### Variables and outcome measures

The primary outcome measure was overall curriculum satisfaction, operationalized as a composite mean score derived from six core components: adequacy of educational resources, graduate autonomy and safety, resident clinical knowledge, clinical-academic balance, research opportunities, and resident professionalism. Secondary outcomes included individual satisfaction scores for each curricular component, identified priority areas for enhancement, and preferences for educational methodologies. Key independent variables examined for their association with these outcomes included professional role (PD versus non-PD), postgraduate training background (local versus international), years of clinical experience, type of institution, and geographic region.

### Statistical analysis

Quantitative data analysis was performed using Statistical Package for the Social Sciences (SPSS) statistical software version 27.0 (International Business Machines [IBM] Corporation). Descriptive statistics, including frequencies, percentages, means, and standard deviations, were calculated to summarize participant characteristics and survey responses. The internal consistency reliability of the composite satisfaction scale was assessed using Cronbach’s alpha (α = 0.81). For demographic comparisons between PDs and non-PDs (Table 1), Fisher’s exact test was used for all categorical variables due to small expected cell frequencies (particularly for PDs, *n* = 9). The Mann-Whitney U test was used for age, which was not normally distributed (Shapiro-Wilk test *p* < 0.05). For bivariate analyses, non-parametric Mann-Whitney U tests were used to compare satisfaction scores between two independent groups (e.g., PDs vs. non-PDs), while Spearman’s rank-order correlation coefficient (ρ) was employed to examine relationships between continuous satisfaction variables. To explore potential predictors of overall curriculum satisfaction, an exploratory multivariable linear regression model was constructed. Given the limited sample size (*n* = 43), we entered only the most theoretically relevant predictors (PD status, international training background, years in practice) to maintain model stability. Age and academic center affiliation were excluded due to limited degrees of freedom. Post‑hoc power analysis indicated that, with *n* = 43 and three predictors, the study had 80% power to detect large effects (Cohen’s f² ≥ 0.35), but not small or moderate effects. Therefore, non‑significant findings should be interpreted cautiously due to the risk of type II error. Model assumptions were verified using residual plots (linearity, homoscedasticity) and the Shapiro–Wilk test for normality of residuals. A two-tailed *p*-value of less than 0.05 was considered statistically significant for all tests.

**Table 1:** Demographic characteristics of survey respondents

Characteristic	Total Cohort (*N* = 43)	Current PDs (*n* = 9)	Non-PDs (*n* = 34)	*p*-value
Age, years (mean ± SD)	44.4 ± 9.5	42.3 ± 7.5	44.9 ± 10.0	0.46†
Gender, n (%)				0.73‡
Male	25 (58.1%)	5 (55.6%)	20 (58.8%)	
Female	18 (41.9%)	4 (44.4%)	14 (41.2%)	
Country of Practice, n (%)				0.22‡
Saudi Arabia	36 (83.7%)	7 (77.8%)	29 (85.3%)	
United Arab Emirates (SCFHS‑accredited program)	7 (16.3%)	2 (22.2%)	5 (14.7%)	
Region (within Saudi Arabia), n (%)				0.35‡
Central	18 (41.9%)	4 (44.4%)	14 (41.2%)	
Western	12 (27.9%)	3 (33.3%)	9 (26.5%)	
Eastern	6 (14.0%)	1 (11.1%)	5 (14.7%)	
Other (including Northern, Southern, and UAE)	7 (16.3%)	1 (11.1%)	6 (17.6%)	
Institution Type, n (%)				0.21‡
Government Hospital	20 (46.5%)	3 (33.3%)	17 (50.0%)	
University/Academic Hospital	15 (34.9%)	4 (44.4%)	11 (32.4%)	
Specialized/Private Center	8 (18.6%)	2 (22.2%)	6 (17.6%)	
Primary Specialty, n (%)				0.09‡
Pediatric Neurology	38 (88.4%)	9 (100%)	29 (85.3%)	
Pediatrics	5 (11.6%)	0 (0%)	5 (14.7%)	
Training Background, n (%)				0.03‡
Saudi Arabia	22 (51.2%)	3 (33.3%)	19 (55.9%)	
North America/Europe	21 (48.8%)	6 (66.7%)	15 (44.1%)	

Appendix: †Mann-Whitney U test; ‡Fisher’s exact test (used due to small cell counts, especially for PDs). Regions within Saudi Arabia are based on SCFHS administrative divisions. ‘Other’ includes Northern and Southern Province (collapsed due to small respondent numbers) in addition to UAE-based respondents. The five respondents with primary specialty ‘Pediatrics’ are general pediatricians with neurology fellowships

Inductive thematic analysis, following the structured approach described by Braun and Clarke, was applied to the free-text responses from open-ended questions. Two researchers independently engaged in an iterative process of data familiarization, systematic code generation, and theme development. The researchers then collaborated to review, refine, and define the final thematic framework. Any discrepancies in coding or theme identification were resolved through discussion until consensus was achieved. We selected representative quotations to illustrate and substantiate the key themes that emerged from the data.

Integration occurred during interpretation, where quantitative results (mean satisfaction scores, correlations, regression) were compared with qualitative themes (e.g., protected time, structured training). Convergence (e.g., low satisfaction with clinical‑academic balance supported by comments on service demands) and expansion (qualitative themes adding nuance not captured quantitatively) are presented in the discussion.

### Ethical considerations

The study protocol was reviewed and approved by the Research Ethics Committee (REC), Faculty of Medicine, King Abdulaziz University, Jeddah, Saudi Arabia, which is registered with the National Committee of Bioethics (NCBE) (Registration No. HA-02-J-008). Ethical approval was granted for the study with reference number 33 − 23 as a non-interventional cross-sectional study. Participation in the study was voluntary. The introductory page of the electronic questionnaire provided detailed information about the study objectives, confidentiality, and participant rights. Completion and submission of the questionnaire were considered implied informed consent. The study was conducted in accordance with the Declaration of Helsinki and Good Clinical Practice (GCP) guidelines [14]. All responses were collected anonymously; no personally identifiable information was obtained. Data were stored securely and analyzed in aggregate form to ensure participant confidentiality and data protection.

## Results

### Participant characteristics

A total of 43 pediatric neurology faculty members from SCFHS‑accredited programs participated in this curriculum evaluation study. The response rate among eligible and available PDs was 90% (9 of 10). A precise response rate for non‑PD faculty could not be calculated because an exact denominator was unavailable due to the purposive sampling approach. As detailed in Table 1, nine participants (20.9%) were current PDs. Of the 43 respondents, 36 (83.7%) were from institutions in Saudi Arabia, while 7 (16.3%) were from SCFHS-accredited training programs in the UAE. Within Saudi Arabia, regional distribution included Central 41.9%, Western 27.9%, Eastern 14.0%, and other regions (Northern, Southern, and UAE) 16.3%. Institution types were balanced, where 46.5% of participants were from government hospitals, 34.9% from university/academic hospitals, and 18.6% from specialized centers, with no significant difference between PDs and non-PDs (*p* = 0.21). A significantly higher proportion of PDs had completed their highest training in North America or Europe compared to non-PDs (66.7% vs. 44.1%, *p* = 0.03).

### Curriculum component satisfaction and perceived adequacy

Faculty perceptions of the current pediatric neurology training curriculum revealed distinct areas of strength and concern (Table 2). Resident professionalism received the highest satisfaction rating (mean 3.8 ± 0.9), with 60.5% of respondents expressing satisfaction. In contrast, the balance between clinical service and academic teaching emerged as the most significant concern, with a mean rating of 2.8 ± 1.3 and 46.5% of faculty expressing dissatisfaction. Research opportunities were also perceived as inadequate by many respondents, with a mean satisfaction score of 2.9 ± 1.2.

**Table 2:** Faculty perceptions of curriculum components (1–5 Likert scale, 5 = very satisfied)

Curriculum Component	Mean Score ± SD	Dissatisfied* *n* (%)	Neutral *n* (%)	Satisfied* *n* (%)	Key Qualitative Themes from Open-Ended Responses
Educational Resources Adequacy	3.4 ± 1.1	11 (25.6%)	13 (30.2%)	19 (44.2%)	• Variable access to simulation equipment • Need for updated neuroimaging resources • Request for standardized online modules
Graduate Autonomy & Safety	3.6 ± 1.1	8 (18.6%)	11 (25.6%)	24 (55.8%)	• Graduates generally prepared for independent practice • Concerns about rare procedure exposure • Adequate supervision during training
Resident Clinical Knowledge	3.6 ± 1.0	6 (14.0%)	17 (39.5%)	20 (46.5%)	• Strong foundational knowledge • Gaps in rapidly evolving subspecialties • Variable exposure to complex cases
Clinical-Academic Balance	2.8 ± 1.3	20 (46.5%)	8 (18.6%)	15 (34.9%)	• Service demands limit academic time • Need protected research periods • Excessive documentation burden
Research Opportunities	2.9 ± 1.2	18 (41.9%)	12 (27.9%)	13 (30.2%)	• Limited dedicated research time • Variable mentorship availability • Infrastructure challenges
Resident Professionalism	3.8 ± 0.9	4 (9.3%)	13 (30.2%)	26 (60.5%)	• Generally strong ethical conduct • Good patient-family communication • Reliable and accountable

Appendix: * Dissatisfied = ratings 1–2; Satisfied = ratings 4–5. Note: High satisfaction with resident professionalism refers to observed behaviors of current trainees. The need for more emphasis on communication/professionalism (Table 3) refers to the desire for additional formal curriculum content, not dissatisfaction with current resident performance

### Identified curriculum gaps and training priorities

Faculty identified specific clinical domains and skills requiring greater emphasis in the current curriculum (Table 3). Genetics and hereditary diseases were most frequently cited as needing more emphasis (74.4%), followed by neuroimmunology (69.8%) and neurodevelopmental disorders (62.8%). When asked which skills required more emphasis within the curriculum (i.e., more structured teaching), research and scholarly activity was most frequently cited (67.4%), followed by communication and professionalism skills (62.8%). Although resident professionalism received high satisfaction ratings (Table 2), a substantial proportion of respondents nevertheless indicated a need for increased curricular emphasis on communication and professionalism training.

**Table 3:** Identified curriculum gaps and training priorities

Domain	Percentage Identifying Need for More Emphasis	Most Commonly Suggested Interventions
Clinical Domains Needing Emphasis		
Genetics & Hereditary Diseases	74.4%	• Dedicated rotation in genetics • Workshops on genomic interpretation • Case-based learning on neurogenetic syndromes
Neuroimmunology	69.8%	• Standardized protocols for autoimmune encephalitis • Exposure to biological therapies • Multidisciplinary neuroimmunology clinics
Neurodevelopmental Disorders	62.8%	• Structured ADHD/autism assessment training • Collaboration with developmental pediatrics • School consultation experience
Skills Requiring More Emphasis		
Research & Scholarly Activity	67.4%	• Protected research time blocks • Structured methodology training • Dedicated research mentorship
Communication & Professionalism	62.8%	• Formal communication skills workshops • Ethics case discussions • Interprofessional team training
Procedural/Technical Skills	52.4%	• Simulation-based electroencephalogram/electromyography training with rotation in the neurophysiology lab with high-volume electroencephalogram/nerve conduction study procedures and experienced mentors, online courses • Ultrasound-guided procedures • Increased lumbar puncture opportunities and simulation-based lumbar puncture training

### Bivariate analyses: Factors influencing curriculum perceptions

Significant correlations emerged between different aspects of curriculum satisfaction, as shown in Table 4. Satisfaction with educational resources was positively associated with satisfaction with research opportunities (ρ = 0.58, *p* < 0.001). Graduate autonomy was positively associated with resident professionalism (ρ = 0.52, *p* < 0.001). These associations are correlational and do not imply causation. Graduate autonomy correlated significantly with resident professionalism (ρ = 0.52, *p* < 0.001). PDs reported significantly lower satisfaction with the clinical-academic balance compared to non-PDs (mean 2.3 vs. 3.0, *p* = 0.04, Mann-Whitney U test).

**Table 4:** Correlation matrix of curriculum perception variables (Spearman’s ρ)

Variable	1	2	3	4	5	6
1. Educational Resources Adequacy	1.00					
2. Graduate Autonomy & Safety	0.48**	1.00				
3. Resident Clinical Knowledge	0.37*	0.41*	1.00			
4. Clinical-Academic Balance	0.43**	0.52**	0.38*	1.00		
5. Research Opportunities	0.58***	0.41*	0.29	0.47**	1.00	
6. Resident Professionalism	0.37*	0.52**	0.44**	0.44**	0.39*	1.00

Appendix: *p < 0.05, **p < 0.01, ***p < 0.001

Faculty with international training background expressed significantly greater concern about research opportunities compared to locally-trained faculty (mean 2.6 vs. 3.2, *p* = 0.03). No significant differences were found based on institution type, years in practice, or gender for overall curriculum satisfaction.

### Multivariable regression: Predictors of overall curriculum satisfaction

A linear regression model was constructed to identify factors predicting overall curriculum satisfaction (composite of 6 curriculum components, Cronbach’s α = 0.81). As shown in Table 5, after controlling for covariates, PD status emerged as a significant negative predictor of satisfaction (β=-0.37, *p* = 0.02), while international training background showed a non-significant positive trend (β = 0.22, *p* = 0.11). The model explained 29% of the variance in overall satisfaction (R²=0.29, Adjusted R²=0.23, F = 4.98, *p* = 0.005).

**Table 5:** Multivariable linear regression: Predictors of overall curriculum satisfaction

Predictor	β Coefficient	95% CI	Standardized β	*p*-value
PD Status	-0.89	(-1.64, -0.14)	-0.37	0.02
International Training	0.52	(-0.12, 1.16)	0.22	0.11
Years in Practice	0.02	(-0.02, 0.06)	0.14	0.28
Constant	3.45	(2.38, 4.52)	-	< 0.001

Model statistics: R² = 0.29, Adjusted R² = 0.23, F = 4.98, p = 0.005

### Educational method preferences and effectiveness

Faculty identified bedside teaching and ward rounds as the most effective educational method (88.4% endorsement), followed by case-based discussions (83.7%) and simulation training (44.2%). Journal clubs were valued by 39.5% of respondents, whereas only 14.0% found didactic lectures highly effective. When rating current resident effectiveness, communication with patients and families received a mean score of 3.6 ± 1.1, while communication with multidisciplinary teams scored 3.5 ± 1.0. Residents’ ability to balance clinical service with patient safety advocacy was rated as average by most respondents (58.1%).

### Qualitative thematic analysis

Following Braun and Clarke’s six-phase approach (familiarization, coding, theme generation, review, definition, and write-up), two researchers independently coded all free-text responses (*n* = 38 respondents provided qualitative comments). Through iterative discussion, three overarching themes were constructed, each with constituent subthemes, as described below.

### Theme 1: The structural tension between service and education

This theme captured the pervasive tension that faculty perceive between clinical service obligations and educational time. Fourteen respondents (32.6%) explicitly mentioned this tension. Subthemes included:

- Erosion of academic time: “Service demands limit academic time” and “excessive documentation burden” were recurrent phrases. One PD elaborated: “Residents spend most of their day on clinical documentation and service tasks, leaving minimal time for reading or research.”
- Need for protected time: Respondents consistently called for “dedicated research blocks,” “elective periods for subspecialty exposure,” and “reduced non-educational service obligations.”
- Consequence identification: Several respondents linked this imbalance to resident burnout and reduced scholarly output. A senior consultant noted: “Without protected time, we cannot expect residents to develop research skills or keep up with rapidly evolving literature.”

### Theme 2: Urgency of subspecialty modernization

Faculty expressed a strong sense that the curriculum has not kept pace with scientific advances. This theme was particularly salient for neurogenetics (12 respondents, 27.9%) and neuroimmunology (10 respondents, 23.3%). Subthemes included:

- Genetics as a foundational competency: Respondents described neurogenetics as “essential for modern practice,” with calls for “workshops on genomic interpretation” and “case-based learning on neurogenetic syndromes.” One respondent stated: “Every pediatric neurologist now needs to interpret next‑generation sequencing reports; this was not part of our own training.”
- Neuroimmunology as an emerging pillar: Requests included “standardized protocols for autoimmune encephalitis,” “exposure to biological therapies,” and “multidisciplinary neuroimmunology clinics.”
- Neurodevelopmental disorders as a persistent gap: While not as “cutting‑edge,” neurodevelopmental disorders (63% citing need) were emphasized for core competence: “Structured ADHD/autism assessment training is lacking in many programs.”

### Theme 3: Infrastructure as a rate-limiting factor

This theme addressed the material and organizational resources needed for effective training. Subthemes included:

- Simulation infrastructure: Nine respondents (20.9%) requested “institutional investment in simulation infrastructure,” specifically for EEG/EMG training, lumbar puncture, and emergency scenarios.
- Standardized educational materials: Seven respondents (16.3%) called for “development of standardized educational materials accessible across institutions,” suggesting a national, shared digital repository.
- Research mentorship: Although not quantified separately, multiple open‑ended responses described “variable mentorship availability” and the need for “dedicated research mentorship” as a structural deficit.
- Representative Quotation: One program director summarized the interplay of all three themes: “Our curriculum must evolve from service-based apprenticeship to structured competency development with protected learning time and modern resources.”
- Theme Development Process: Initial codes (e.g., “service burden,” “research time,” “genetics training,” “simulation access”) were grouped into candidate themes. Themes were reviewed against coded extracts and the full dataset. The final three themes were defined through consensus discussion between the two analysts, with each theme representing a distinct yet interrelated dimension of curriculum inadequacy in structural (Theme 1), content (Theme 2), and resource (Theme 3) domains.

### Summary of key curriculum findings

The pediatric neurology training curriculum in SCFHS-accredited programs demonstrates strengths in developing clinical competence and professionalism. Still, it reveals systemic challenges in three key areas: (1) imbalance between clinical service and academic development, (2) inadequate research infrastructure and opportunities, and (3) gaps in rapidly evolving subspecialty content. PDs, who bear operational responsibility for curriculum implementation, express significantly greater concern about these limitations compared to non-PD faculty.

## Discussion

This multi-institutional evaluation study reveals that while pediatric neurology training within the SCFHS‑accredited system demonstrates robust clinical and professional foundations, it faces significant systemic challenges in research support, academic–service balance, and subspecialty content, concerns that both mirror global trends and reflect region-specific priorities. The high satisfaction with resident professionalism (observed behaviors) is encouraging and aligns with reported outcomes from established North American programs. However, the simultaneous call for more curriculum emphasis on communication and professionalism training (Table 3) indicates that faculty value these skills highly and wish to see them more explicitly and systematically taught, rather than simply expected. This distinction between satisfaction with current resident performance and desire for enhanced formal instruction is important for curriculum planning, where professionalism is increasingly codified within competency frameworks [15]. Nonetheless, the pronounced discontent with clinical-academic balance and research infrastructure presents a critical divergence. This finding mirrors challenges reported in resource-constrained settings and high-volume clinical environments, where service demands consistently encroach upon dedicated educational time [16]. The strong correlation (ρ = 0.58) between perceptions of educational resources and research opportunities underscores a synergistic deficit; without protected time and modern tools, scholarly activity becomes unsustainable, a barrier extensively documented in internal medicine and surgical training programs globally [17].

The identified priority for enhanced training in genetics and neuroimmunology is not merely a local gap but reflects a worldwide educational lag behind rapid scientific advancement. Similar curriculum deficits have been reported in recent needs assessments from European and Asian training boards, indicating a universal struggle to integrate complex, technology-driven subspecialties into standardized curricula [18, 19]. The faculty’s preference for bedside teaching and case-based discussions over didactic lectures is consistent with adult learning theory and resonates with pedagogical shifts in medical education internationally, affirming that clinical reasoning and contextual learning remain paramount even in an era of digital innovation [20].

A key finding of this study is the significantly more critical perspective held by PDs. Their lower satisfaction may reflect an operational vantage point. One possible interpretation is that PDs function as de facto institutional barometers, and their heightened dissatisfaction could be related to first-hand exposure to resource constraints, accreditation pressures, and operational realities that may be less visible to non-PD faculty. However, this interpretation is speculative and should be tested in future research [21]. Furthermore, the trend toward greater concern among internationally trained faculty regarding research infrastructure may reflect exposure to more robust academic environments during their training, establishing higher benchmarks for comparison. This comparative perspective has been observed in other medical systems integrating globally trained physicians, often serving as a catalyst for reform [22].

The regression model identifying PD status as a key predictor of dissatisfaction, accounting for a meaningful portion of variance in overall sentiment, reinforces the concept that curriculum evaluation is perspective-dependent. This finding supports the mandatory inclusion of PD insight in any national training reform committee, as their concerns are empirically linked to first-hand management of the curriculum’s limitations [23]. The gaps identified suggest potential directions for curriculum enhancement. One such direction may be shifting from predominantly service-oriented models toward a more structured competency-based framework based on EPAs. Formal EPA development could, if implemented, allow progressive entrustment decisions, structured assessment tools, and transparent expectations. However, these are exploratory suggestions based on faculty perceptions; further research is needed to evaluate their feasibility and impact in the local context.

Limitations and future directions.

This study has several limitations. Its cross-sectional design captures perceptions at a single point in time and cannot assess causal relationships or trends. The findings represent faculty perceptions rather than an objective evaluation of program structure or outcomes. Therefore, conclusions regarding needed reforms are exploratory and hypothesis‑generating. They should inform future curriculum development and hypothesis‑driven research rather than directly mandate systemic change. The sample size (*n* = 43) is a limitation. While representative of the relatively small specialty community in the region, it limited statistical power for multivariable regression, particularly for detecting small‑to‑moderate effects. Subgroup comparisons (e.g., PDs vs. non‑PDs) should be considered exploratory. Future studies with larger, multi‑regional samples are needed to confirm these findings. The data are self-reported and may be subject to social desirability or recall bias. Also, demographic comparisons in Table 1 used Fisher’s exact test and Mann-Whitney U test to account for small cell counts; however, the small number of PDs (*n* = 9) limits the statistical power to detect small differences between groups. One apparent inconsistency, high satisfaction with resident professionalism yet demand for more emphasis on professionalism training, reflects the difference between assessing current trainee behaviors (satisfaction) and advocating for more structured curriculum content (need for emphasis). This distinction is clarified in the results and discussion. Furthermore, this study did not include the perspectives of residents or recent graduates. Their feedback is essential for a complete curriculum evaluation. Future research should incorporate trainee and graduate perspectives to provide a 360‑degree view of training efficacy. Additionally, it should adopt longitudinal designs to track the impact of specific curriculum interventions. Objective metrics of training outcomes, such as board pass rates, scholarly output, and graduate career trajectories, should be correlated with faculty perceptions to validate these findings.

The first pediatric neurology training program was established in the US in the mid-1950s, and structured curricula have since formed the foundation of residency education. However, the field has evolved rapidly, driven by advances in genetics, immunotherapy, cellular therapies, and artificial intelligence. While maintaining strong core clinical training is essential, residency programs must also adapt to prepare trainees for these emerging developments. Incorporating flexible, dynamic elective rotations can provide residents with structured exposure to high-impact areas such as neurogenetics and precision medicine, gene therapy and gene editing, neuroimmunology, neuro-oncology and cellular therapies, epilepsy innovation, neurocritical care, advanced neuromuscular therapeutics, clinical trials, and artificial intelligence in neurology. Such integration ensures that future pediatric neurologists are not only clinically competent but also equipped to lead in an era increasingly defined by precision and advanced therapeutics.

## Conclusion

This study represents the first comprehensive, faculty-informed multi-institutional assessment of the pediatric neurology training curriculum in the region, providing an evidence base for targeted reform. While perceived strengths of the training system include resident foundational clinical competence and professionalism, faculty (PDs in particular) identify structural challenges related to academic balance, research infrastructure, and integration of rapidly evolving subspecialties. PDs’ heightened dissatisfaction serves as a vital barometer for these systemic pressures. These perception-based findings suggest that addressing these issues may require more than incremental adjustment; they point toward a possible structural re‑imagination of training paradigms. Potential areas for consideration include strategic investment in competency‑based reform, such as defined EPA, simulated learning environments, protected academic time, strengthened research mentorship, and collaborative national educational resources in high‑priority domains like neurogenetics. However, these are exploratory implications derived from faculty perceptions; pilot studies and further evaluation would be needed before implementation. Aligning the training curriculum and assessment systems with clearly articulated competencies, consistent with global benchmarks and responsive to the academic aspirations of faculty, will be essential to ensuring sustainable, high-quality pediatric neurology training that addresses regional healthcare needs while meeting international standards.

## Supplementary Information

Supplementary Material 1.

## Data Availability

The datasets generated and analyzed during the current study are not publicly available to protect the anonymity and confidentiality of the participants but are available from the corresponding author on reasonable request.

## Abbreviations

ACGME: Accreditation Council for Graduate Medical Education
ADHD: Attention-Deficit/Hyperactivity Disorder
CBME: Competency-based medical education
CI: Confidence Interval
EEG: Electroencephalogram
EMG: Electromyogram
EPA: Entrustable Professional Activities
GCP: Good Clinical Practice
NCBE: National Committee of Bioethics
PD: Program Director
REC: Research Ethics Committee
SCFHS: Saudi Commission for Health Specialties
SD: Standard Deviation
SPSS: Statistical Package for the Social Sciences
STROBE: Strengthening the Reporting of Observational Studies in Epidemiology
UAE: United Arab Emirates
