# Supplementary material for: Evaluating the pediatric neurology training curriculum in SCFHS-accredited programs: a multi-institutional survey study
Source: BMC Med Educ. 2026 Jul 17;26:1636. doi: 10.1186/s12909-026-09926-w (PMC13660195; doi:10.1186/s12909-026-09926-w)
Supplement: Supplementary file 1 [file 12909_2026_9926_MOESM1_ESM.pdf]

Perceptions of Pediatric Neurologists and Program Directors on the Ideal Candidate

This is a data collection sheet for study entitled:
Perceptions of Pediatric Neurologists and Program Directors on the Ideal Candidate

Objective:
To evaluate the perceptions of pediatric neurology residency program directors regarding the qualities of the ideal residency candidate

Study design:
Cross-sectional study

Primary center for contact:
King Abdulaziz University

IRB details:
For any concerns please contact the Unit of Biomedical Ethics at King Abdulaziz University (med.rcommittee@kau.edu.sa)

Instructions:
Kindly follow the suggested format in the description of present

Estimated duration:
It is expected that completing the survey will take up to 5 minutes

Contact details:
For any concerns kindly contact: *Anas Alyazidi (alyazidi.anas@gmail.com) - (00966 59 493 1999)*

* تشير إلى أنّ السؤال مطلوب

1. **Do you agree to participate?***

حدد دائرة واحدة فقط.

Yes

Demographic data

This section collects basic demographic details. It helps us understand the diversity of responders and analyze responses across different groups.

2. **Are you currently a program director?*** Dropdown

حدد دائرة واحدة فقط.

- Yes
- No (previous program director)
- No (never been a program director)

3. **Age***

4. **Gender*** Dropdown

حدد دائرة واحدة فقط.

- Male
- Female

5. **Current country of practice*** Dropdown

حدد دائرة واحدة فقط.

- Saudi Arabia
- GCC *التخطي إلى السؤال 7*
- Middle East *التخطي إلى السؤال 8*
- Other/International *التخطي إلى السؤال 9*
- Prefer not to say *التخطي إلى السؤال 10*

Saudi Arabia

This section lists the main tertiary and teaching hospitals in Saudi Arabia that host pediatric neurology services or residency training programs. Please select your current centre if listed.

6. **Center***

Please select the medical center where you currently (or previously) serve as the program director or practice for pediatric neurology. This will help us understand institutional perspectives across different training sites.

Dropdown

حدد دائرة واحدة فقط.

- Dr. Soliman Fakeeh Hospital (group)*التخطي إلى السؤال 10*
- Dr. Sulaiman Al Habib Medical Group*التخطي إلى السؤال 10*
- International Medical Center (IMC)*التخطي إلى السؤال 10*
- King Abdulaziz Medical City (KAMC) — Riyadh*التخطي إلى السؤال 10*
- King Abdulaziz University Hospital (KAUH) — Jeddah*التخطي إلى السؤال 10*
- King Abdullah Specialist Children's Hospital (KASCH) — Riyadh *التخطي إلى السؤال 10*
- King Fahd Hospital of the University (KFHU) — Al Khobar*التخطي إلى السؤال 10*
- King Fahd Medical City (KFMC) — Riyadh*التخطي إلى السؤال 10*
- King Fahd Specialist Hospital / regional tertiary hospitals (Dammam / Tabuk / other)*التخطي إلى السؤال 10*
- King Faisal Specialist Hospital & Research Centre (KFSHRC) — Jeddah *التخطي إلى السؤال 10*
- King Faisal Specialist Hospital & Research Centre (KFSHRC) — Riyadh *التخطي إلى السؤال 10*
- King Khalid University Hospital (KKUH) — Riyadh*التخطي إلى السؤال 10*
- King Saud Medical City (KSMC) — Riyadh*التخطي إلى السؤال 10*
- King Saud University Medical City (KSUMC) / KSU Hospital — Riyadh *التخطي إلى السؤال 10*
- Ministry of National Guard Health Affairs (MNGHA / NGHA) — Riyadh / regional sites*التخطي إلى السؤال 10*
- Prince Sultan Military Medical City (PSMMC) — Riyadh*التخطي إلى السؤال 10*
- Security Forces Hospital (SFH) — Riyadh*التخطي إلى السؤال 10*
- Other*التخطي إلى السؤال 10*

GCC

This section includes major centres in the Gulf Cooperation Council (GCC) countries outside Saudi Arabia (UAE, Qatar, Oman, Kuwait, Bahrain). Select your institution if you are based in one of these hospitals.

7. **Center***

Please select the medical center where you currently (or previously) serve as the program director or practice for pediatric neurology. **In case your exact center isn't mentioned, please choose the option with your country of practice.** This will help us understand institutional perspectives across different training sites.

Dropdown

حدد دائرة واحدة فقط.

- Bahrain — Salmaniya Medical Complex*التخطي إلى السؤال 10*
- Kuwait — Jaber Al-Ahmad Hospital / Mubarak Al-Kabeer Hospital / Kuwait University Hospital*التخطي إلى السؤال 10*
- Oman — Sultan Qaboos University Hospital / Royal Hospital (Muscat) *التخطي إلى السؤال 10*
- Qatar — Sidra Medicine (Doha) / Hamad Medical Corporation (Women's & Children's)*التخطي إلى السؤال 10*
- United Arab Emirates — Al Jalila Children's Specialty Hospital (Dubai) *التخطي إلى السؤال 10*
- United Arab Emirates — Tawam Hospital (Al Ain) / Johns Hopkins Medicine Abu Dhabi partner sites*التخطي إلى السؤال 10*
- United Arab Emirates — Sheikh Khalifa Medical City / Cleveland Clinic Abu Dhabi *التخطي إلى السؤال 10*

Other (non-GCC Middle East)

This section covers pediatric neurology centres across the wider Middle East region (e.g., Jordan, Lebanon, Egypt, Iraq, Turkey). Choose this option if you are affiliated with a Middle Eastern institution outside the GCC. **In case your exact center isn't mentioned, please choose the option with your country of practice.**

8. **Center***

Please select the medical center where you currently (or previously) serve as the program director for pediatric neurology. This will help us understand institutional perspectives across different training sites.

Dropdown

حدد دائرة واحدة فقط.

- Egypt — (e.g., Cairo University Children's Hospital / Ain Shams) *التخطي إلى السؤال 10*
- Iraq / Palestine / Syria / Yemen / Turkey — (regional tertiary centres) *التخطي إلى السؤال 10*
- Jordan — (e.g., King Hussein Medical Center / Jordan University Hospital) *التخطي إلى السؤال 10*
- Lebanon — (e.g., American University of Beirut Medical Center) *التخطي إلى السؤال 10*
- Other*التخطي إلى السؤال 10*

Other (non-Middle East / International)

Use this option if you are affiliated with a hospital or institution outside the Middle East region.

9. **Center***

Please select the medical center where you currently (or previously) serve as the program director or practice for pediatric neurology. This will help us understand institutional perspectives across different training sites.

____

Demographic data (cont.)

This section collects basic demographic details. It helps us understand the diversity of responders and analyze responses across different groups.

10. **Type of your current institution*** Dropdown

حدد دائرة واحدة فقط.

- University/Academic Hospital
- Specialized Tertiary Care Center
- Government Hospital
- Military Hospital
- National Guard Health Affairs
- Private Hospital

11. **Which country did you complete your training?***

Please indicate the country where you completed your formal residency and/or fellowship training. This information will help us explore how international versus local training backgrounds may influence perspectives on residency candidate selection. Dropdown

حدد دائرة واحدة فقط.

- Saudi Arabia
- Canada
- United States
- United Kingdom
- Europe
- Australia
- East Asia
- GCC country (other than Saudi Arabia)
- Arab country (other than the GCC countries)

12. **Was your previous selection for your residency or fellowship training?*** Dropdown

حدد دائرة واحدة فقط.

- I selected the country of my residency training
- I selected the country of my fellowship training
- Both

13. **Years as a program director*** Dropdown

حدد دائرة واحدة فقط.

- 0 (Never been a program director)
- 1
- 2
- 3
- 4
- 5
- 6
- 7
- 8
- 9
- 10
- 11
- 12
- 13
- 14
- 15
- 16
- 17
- 18
- 19
- 20
- 21
- 22
- 23
- 24
- 25
- 26
- 27
- 28
- 29
- 30
- >30

14. **Your main specialty background*** Dropdown

حدد دائرة واحدة فقط.

- Pediatric neurology
- Pediatrics
- Neurology
- Other

General Selection Criteria

Rank the importance of the following factors in selecting candidates.

Likert scale: Not Important [1] → Very Important [5]

15. **Academic performance (GPA)***

حدد دائرة واحدة فقط.

	1	2	3	4	5	
Not						Very important

16. **Academic performance (SMLE score)***

حدد دائرة واحدة فقط.

	1	2	3	4	5	
Not						Very important

17. **Clinical rotations performance (especially neurology & pediatrics)***

حدد دائرة واحدة فقط.

	1	2	3	4	5	
Not	◯	◯	◯	◯	◯	Very important

18. **Professionalism & attitude***

حدد دائرة واحدة فقط.

	1	2	3	4	5	
Not	◯	◯	◯	◯	◯	Very important

19. **Letters of recommendation***

حدد دائرة واحدة فقط.

	1	2	3	4	5	
Not	◯	◯	◯	◯	◯	Very important

20. **Interview performance***

حدد دائرة واحدة فقط.

	1	2	3	4	5	
Not	◯	◯	◯	◯	◯	Very important

21. **Communication skills***

حدد دائرة واحدة فقط.

	1	2	3	4	5	
Not	◯	◯	◯	◯	◯	Very important

22. **Long-term career plans***

حدد دائرة واحدة فقط.

	1	2	3	4	5	
Not	◯	◯	◯	◯	◯	Very important

23. **Research experience (number, quality, dissemination)***

حدد دائرة واحدة فقط.

	1	2	3	4	5	
Not	◯	◯	◯	◯	◯	Very important

24. **Leadership and extracurricular activities***

حدد دائرة واحدة فقط.

	1	2	3	4	5	
Not	◯	◯	◯	◯	◯	Very important

25. **Which factor do you personally consider most important for acceptance?** *

حدد كل الإجابات الملائمة.

- [ ] Academic performance (GPA)
- [ ] Academic performance (SMLE score)
- [ ] Clinical rotation performance (especially pediatrics/neurology)
- [ ] Professionalism and attitude
- [ ] Letters of recommendation
- [ ] Interview performance
- [ ] Communication skills
- [ ] Long-term career plans
- [ ] Research experience
- [ ] Leadership and extracurricular activities
- [ ] أخرى: ____________________

Specific to Pediatric Neurology

Likert scale: Not Important [1] → Very Important [5]

26. **How important is prior exposure to pediatric neurology (electives, observerships, rotations)?** *

حدد دائرة واحدة فقط.

	1	2	3	4	5	
Not	○	○	○	○	○	Very important

27. **How important is demonstrated interest in pediatric neurology (courses, electives, student clubs)?** *

حدد دائرة واحدة فقط.

	1	2	3	4	5	
Not	○	○	○	○	○	Very important

28. **Do you value applicants who have participated in multidisciplinary care settings (rehab, epilepsy, NICU)?** * Dropdown

.حدد دائرة واحدة فقط

Yes

No

Research-Related Preferences

Likert scale: Not Important [1] → Very Important [5]

29. **How important is applicant involvement in research?***

.حدد دائرة واحدة فقط

	1	2	3	4	5	
Not						Very important

30. **Which type(s) of research do you value most?***

.حدد كل الإجابات الملائمة

Clinical research
Basic science / laboratory research
Quality improvement projects
Medical education research
Community or public health research
Translational research
No specific preference

:أخرى

31. **Which research study designs do you consider most valuable in evaluating a candidate's academic experience?** *

حدد كل الإجابات الملائمة.

- [ ] Case report / Case series
- [ ] Cross-sectional study
- [ ] Case-control study
- [ ] Cohort study
- [ ] Randomized controlled trial (RCT)
- [ ] Systematic review / Meta-analysis
- [ ] Quality improvement project
- [ ] No specific preference
- [ ] أخرى: ____________________

32. **Which research outputs do you value?** *

حدد كل الإجابات الملائمة.

- [ ] First-author publication
- [ ] Any publication (regardless of author order)
- [ ] Poster/oral presentation at conference
- [ ] Ongoing project with strong methodology
- [ ] Research awards / recognitions

33. **In your opinion, meaningful research experience reflects which applicant traits?** *

حدد كل الإجابات الملائمة.

- [ ] Intellectual curiosity
- [ ] Critical & analytical thinking
- [ ] Self-directed learning
- [ ] Commitment to specialty
- [ ] Academic career interest
- [ ] أخرى: ____________________

34. **Optional:**

In your own words, describe the top three qualities of the ideal pediatric neurology resident?

Perceptions on Trainee Education & Program Needs

This section aims to explore your views on the current strengths, needs, and expectations for **pediatric neurology residents** during training. Your responses will help identify opportunities to improve education, clinical exposure, and overall program quality.

35. **How satisfied are you with the current educational resources available for trainees?** * Dropdown

.حدد دائرة واحدة فقط

- Very satisfied
- Satisfied
- Neutral
- Dissatisfied
- Very dissatisfied

36. **How satisfied are you with the degree of autonomy and safety to practice independently demonstrated by your program's graduates at the end of training?** * Dropdown

حدد دائرة واحدة فقط.

Very satisfied
Satisfied
Neutral
Dissatisfied
Very dissatisfied

37. **In your opinion, which skills require more emphasis in residency training?** *

حدد كل الإجابات الملائمة.

- [ ] Clinical knowledge in pediatric neurology
- [ ] Procedural/technical skills (EEG, EMG, etc.)
- [ ] Research and scholarly activity
- [ ] Communication and professionalism
- [ ] Leadership and teamwork
- [ ] Patient safety and quality improvement

38. **How satisfied are you with residents' current level of clinical knowledge and diagnostic skills in pediatric neurology?** * Dropdown

حدد دائرة واحدة فقط.

Very satisfied
Satisfied
Neutral
Dissatisfied
Very dissatisfied

39. **Which clinical domains require more emphasis?** *

حدد كل الإجابات الملائمة.

- [ ] Epilepsy & EEG
- [ ] Neurodevelopmental disorders
- [ ] Neuromuscular disorders
- [ ] Neuroimmunology
- [ ] Neurocritical care (PICU/Status epilepticus)
- [ ] Genetics and hereditary diseases
- [ ] General pediatric neurology exposure

40. **How adequate is the current balance between clinical service and academic teaching?** *

Dropdown

حدد دائرة واحدة فقط.

- Very satisfied
- Satisfied
- Neutral
- Dissatisfied
- Very dissatisfied

41. **Which educational methods do you find most effective for trainees?** *

حدد كل الإجابات الملائمة.

- [ ] Bedside teaching / ward rounds
- [ ] Didactic lectures
- [ ] Case-based discussions
- [ ] Simulation training
- [ ] Research mentorship
- [ ] Journal clubs
- [ ] أخرى: ____________

42. **How effective are residents in communication with patients/families** *

Likert scale: Not effective [1] → Very effective [5]

حدد دائرة واحدة فقط.

	1	2	3	4	5	
Not	◯	◯	◯	◯	◯	Very effective

43. **How effective are residents in communication with multidisciplinary teams** *

Likert scale: Not effective [1] → Very effective [5]

حدد دائرة واحدة فقط.

	1	2	3	4	5	
Not	◯	◯	◯	◯	◯	Very effective

44. **How well do residents balance clinical service with advocacy for patient safety and quality of care?** * Dropdown

حدد دائرة واحدة فقط.

- Excellent
- Good
- Average
- Fair
- Poor

45. **Do you think residents should receive structured training in:***

حدد كل الإجابات الملائمة.

- [ ] Leadership & management
- [ ] Patient safety & quality improvement
- [ ] Health advocacy & community engagement
- [ ] Research methodology & evidence-based medicine
- [ ] Medical education & teaching skills
- [ ] Ethics, professionalism, and communication

46. **How adequate are current opportunities for residents in research and scholarly activity?** * Dropdown

حدد دائرة واحدة فقط.

- Very adequate
- Adequate
- Neutral
- Inadequate
- Very inadequate

47. **How satisfied are you with residents' professionalism (ethics, responsibility, accountability)?** * Dropdown

حدد دائرة واحدة فقط.

- Very adequate
- Adequate
- Neutral
- Inadequate
- Very inadequate

48. **Which domains of research do you consider most valuable in evaluating a candidate?** *

حدد كل الإجابات الملائمة.

- [ ] Clinical research
- [ ] Quality improvement projects
- [ ] Case reports & case series
- [ ] Medical education research
- [ ] Systematic reviews/meta-analyses
- [ ] أخرى: _______________

49. **Optional:**

What are your top expectations from residents during their training?

____

50. **Optional:**

What additional resources, structural changes, or innovations do you recommend to better support residents' training and career development?

____

____

____

____

____

لم يتم إنشاء هذا المحتوى ولا اعتماده من قبل Google.

Google نماذج
